# Using the RDP Classifier to Predict Taxonomic Novelty and Reduce the Search Space for Finding Novel Organisms

**DOI:** 10.1371/journal.pone.0032491

**Published:** 2012-03-05

**Authors:** Yemin Lan, Qiong Wang, James R. Cole, Gail L. Rosen

**Affiliations:** 1 School of Biomedical Engineering, Science, and Health Systems, Drexel University, Philadelphia, Pennsylvania, United States of America; 2 Ribosomal Database Project, Michigan State University, East Lansing, Michigan, United States of America; 3 Department of Electrical and Computer Engineering, Drexel University, Philadelphia, Pennsylvania, United States of America; Argonne National Laboratory, United States of America

## Abstract

**Background:**

Currently, the naïve Bayesian classifier provided by the Ribosomal Database Project (RDP) is one of the most widely used tools to classify 16S rRNA sequences, mainly collected from environmental samples. We show that RDP has 97+% assignment accuracy and is fast for 250 bp and longer reads when the read originates from a taxon known to the database. Because most environmental samples will contain organisms from taxa whose 16S rRNA genes have not been previously sequenced, we aim to benchmark how well the RDP classifier and other competing methods can discriminate these novel taxa from known taxa.

**Principal Findings:**

Because each fragment is assigned a score (containing likelihood or confidence information such as the boostrap score in the RDP classifier), we “train” a threshold to discriminate between novel and known organisms and observe its performance on a test set. The threshold that we determine tends to be conservative (low sensitivity but high specificity) for naïve Bayesian methods. Nonetheless, our method performs better with the RDP classifier than the other methods tested, measured by the f-measure and the area-under-the-curve on the receiver operating characteristic of the test set. By constraining the database to well-represented genera, sensitivity improves 3–15%. Finally, we show that the detector is a good predictor to determine novel abundant taxa (especially for finer levels of taxonomy where novelty is more likely to be present).

**Conclusions:**

We conclude that selecting a read-length appropriate RDP bootstrap score can significantly reduce the search space for identifying novel genera and higher levels in taxonomy. In addition, having a well-represented database significantly improves performance while having genera that are “highly” similar does not make a significant improvement. On a real dataset from an Amazon Terra Preta soil sample, we show that the detector can predict (or correlates to) whether novel sequences will be assigned to new taxa when the RDP database “doubles” in the future.

## Introduction

The 16S ribosomal RNA (rRNA) gene has become a standard in microbial community surveys [1], [2], [3], [4], [5]. While the 16S rRNA gene is the standard for identification of bacterial isolates and the discovery of novel bacteria [6], 16S rRNA has its faults [7]. Not all known species can be resolved with 16S rRNA, and it is impossible to use 16S sequences to discriminate strains – the maximum difference between members of the same species is very small, approximately 1–1.5% sequence difference for complete gene sequences [8]. For example, in a study of 683 isolates obtained from clinical specimens, 83% were able to be resolved at species level and 99% at the genus- level [9]. Due to this sensitivity of the 16S rRNA gene, the ribosomal database project (RDP) [10] offers genus-level classification through a naïve Bayes classifier [11].

The RDP 16S rRNA classifier has become a standard way that biologists, ecologists, and clinicians identify full-length and sub-sequences of 16S rRNA sequences and is widely cited under a wide range of applications. Scientists have used the RDP classifier to a) correlate mammals to their gut microbes [12], b) correlate microbiota composition to human obesity [13], c) study the soil community structure [14], and d) investigate diversity of surface ocean waters [15]. The unique advantage of the RDP classifier is that it not only provides the best matching taxa but offers a bootstrap confidence score, which is able to give a level of confidence to the assignment it makes. The bootstrap score will give low confidence to a read if it does not match its assignment well. Most likely, if a poor assignment is made, this is due to the read originating from an organism not in the database. Therefore, the RDP classifier may be able to determine novel taxa at various phylogenetic levels, but no study has benchmarked how well it performs this task.

We aim to study the RDP classifier bootstrap score against recently proposed methods that can be used for a similar purpose. NBC is a variant of the naïve Bayes classifier that can also be applied to whole genome sequences) [16], Phymm(BL) is another method originally proposed for whole-genome sequences but can be used on any database [17], and PhylOTU [18] which is an alignment-based clustering designed for 16S rRNA sequences.

The question remains – what is novelty? Novelty in this paper is defined as a sequence that forms a new clade with respect to a particular taxonomic level, or a clade that is not nested within a clade of previously sequenced organisms. In other words, a novel taxon is one that has no representatives in the database. An example is that we may find that the bootstrap score is confident for the Enterobacteriales clade but has low-confidence for the Escherichia genus. In this case, we would deduce that this read derives from a novel genus within Enterobacteriales (where Escherichia happens to be its “closest” relative in the database, where closeness is relative). In our studies, we do not claim to be able to bin reads that may originate from a similar novel taxon, but just to be able to discriminate a 16S rRNA sequence representing a new taxon from known lineages in the database.

In our work, we demonstrate that the RDP classifier combined with a detector (trained with a bootstrap threshold) performs the best for 500 bp reads. In addition, we demonstrate the RDP classifier+detector on a real soil dataset and show that the detector predicts novel genera (e.g. low-confidence reads with a small database are more likely to match better to newer taxa in a larger database). We can combine our detector with most other composition-based taxonomic classifiers and do so when benchmarking performance. Due to this restriction, we only use the terminology of RDP classifier in this paper, and we use RDP and RDP classifier interchangeably.

### I. Background on detection theory and experimental design for supervised learning

In supervised classification, experiments are usually limited by the amount of data available. In order to test how well a method works, a part (usually majority) of the data is used to train a classifier. The part of the data used for training is the training set while the part left out of the training is the test set. If one were to do one iteration like this, it can give an idea of how the classifier will perform when it sees never-seen-before data. To develop an idea for how its performance may vary on new data, 5-fold cross-validation is performed, which usually takes random 4/5ths of the data and test on the other 1/5th.

In detection theory, a classifier is a way to map observations of an event into a class by scoring an input. If we can use a two-class system, such as classifying something as known or novel, then we have a “positive” (in our case, a positive is something that is known in our database) and a “negative” (a read from a novel organism). A detector tries to identify the positive from negative classes. When classifying in this manner, we have four different scenarios – a true positive (TP) which is a read from a known organism that is correctly classified as a known, a false negative (FN) which is a read from a known organism falsely classified as novel, a true negative (TN) which is a read form a novel organisms correctly classified as novel, and a false positive (FP) which is a read from a novel organism falsely classified as a known. Ideally, a classifier will have many TPs and TNs and attempt to minimize the FPs and FNs. But depending on the classifier, one can have more FNs than FPs which would mean that many reads from known taxa are getting classified as novel but the reads from novel are rarely misclassified. Therefore, there are two types of error (Type I and Type II).

Based on these different rates, a receiver operating characteristic (ROC) curve can be constructed that illustrates the true positive rate (TPR is sensitivity) vs. the false positive rate (FPR is 1-specificity). Given the TPs, FPs, FNs, and TNs, one can choose the threshold that attempts to maximize the TPR while minimizing the FPR. In our work, we choose the threshold that maximizes the harmonic mean of the sensitivity and specificity. We noticed that this threshold is not always consistent and will depend on the subset chosen. Therefore, we decided to break a detector training set up into folds (as in cross validation), and to average threshold over 5 folds. This is just the training step and would still need data left out as “test” (in this case we decide to do a half-fold for training and testing the detector).

## Methods

### I. Construction of datasets

#### a. RDP Training Data version 6

For the full database, we acquired RDP Classifier's TrainingData6 from the associated website. This set contains 8422 sequences belonging to 1712 genera, 311 families, 112 orders, 79 classes, 39 phyla, and 2 kingdoms.

**Figure 1:**
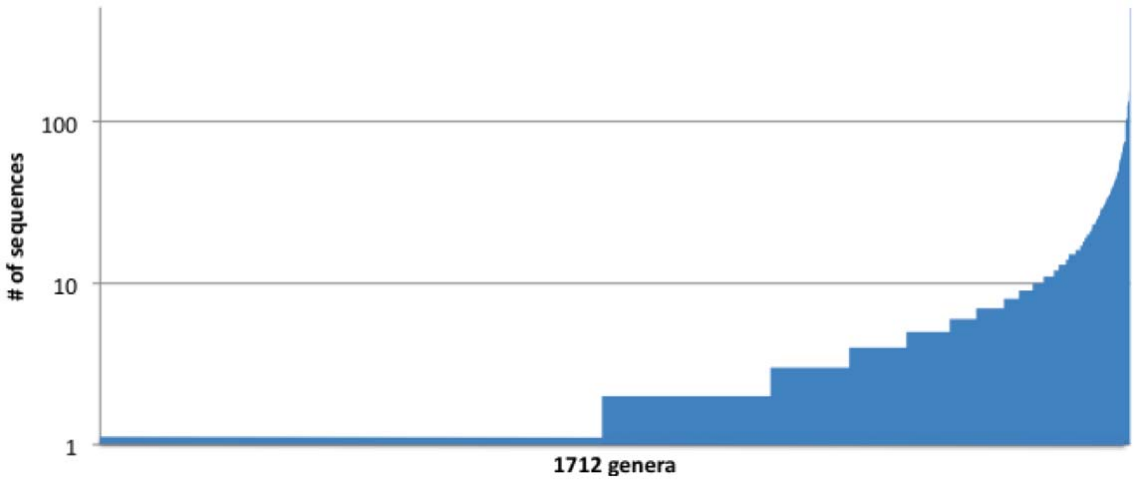
The number of sequences per genera (log-scale) demonstrating the imbalance of the database.

Fig. 1 demonstrates the unbalanced nature of the database. Out of the ∼8000 sequences, about 50% of the genera contain one example sequence, because for many genera, there is only a single known (named) species. Whereas on the other extreme, Streptomyces has 508 sequences from 508 different species and subspecies, and is the most well-represented genus.

#### b. Half-fold experiment using all sequences

**Figure 2:**
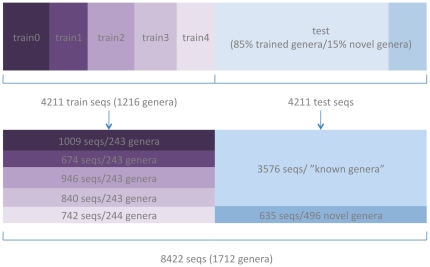
Setup for the “half-fold” experiments where half the sequences were used for training and half for testing.

For most experiments (except for investigations into well-represented and highly-similar genera), we constructed a standard dataset for training the detector using half of the 16S rRNA sequences, and a test dataset formed from the other half of the sequences (sequence distribution illustrated in Fig. 2, Table 1, and known/novel distribution illustrated in Table 2). The detector-training half of the dataset was used to train the detector threshold. Once the detector threshold is determined (through 5-fold experiments), we can test its feasibility on the test dataset (the other half of the data). In order to train the detector threshold, we trained the classifier database on 1/5 of the detector-training data on each iteration and left the other 4/5 (of the detector-training data) as novel data. This is different from 5-fold experiments where one uses “most” of the data for training data (commonly using 4/5 for training and 1/5 for testing). In our case, we're using 4/5 novel data and 1/5 known data. We chose this experimental design with the assumption that we want to train a threshold that is accustomed to having 20% known and 80% novel data, which we believe is more reflective of diverse environments such as soil, where ∼95% of the organisms may be novel. We did not want to train a detector threshold that was used to a majority of the data being represented in the training database.

**Table 1:** The taxa breakdown when testing on all the taxonomic levels.

4211 training seqs	4211 testing seqs	
1216 genera	486 genera novel	673 genera known
272 families	39 families novel	228 families known
101 orders	11 orders novel	85 orders known
76 classes	3 classes novel	70 classes known
37 phyla	2 phyla novel	33 phyla known

*While genera have 29% novel representation in the test set (in 15% of the sequences mentioned in* *Fig. 2* *), 14.3% of the families are novel (in 15% of the sequences), 11% of the orders are novel (in 5% of the sequences), 4% of the classes are novel (in 2.4% of the sequences), and 5% of the phyla are novel (in 0.07% of the sequences).*

**Table 2:** The number of known/novel reads on genus level selected per training set for use in detector design and the separate test set.

Half-fold	Training1	Training2	Training3	Training4	Training5	Testing
**100 bp**	12150/48650	12150/48650	12150/48650	12150/48650	12200/48600	35750/6350
**250 bp**	4840/19440	4860/19420	4840/19440	4860/19420	4880/19400	14268/2536
**500 bp**	2380/9580	2380/9580	2380/9580	2410/9550	2410/9550	6956/1238

Therefore, the training dataset was split in a 5-fold fashion to train a detector threshold that can discriminate between reads of known and novel origin. Each 5-fold split was selected to contain nearly 1/5 of the genera in the training dataset. There are no novel taxa at the family-level or higher in the test dataset compared to the training dataset. Subsequently, a read dataset was constructed from the entire dataset to determine a threshold for this training dataset, (Table 2.) In order to test each rank higher than genus, a separate dataset was constructed (Table 3).

**Table 3:** The known/novel training and testing dataset composition when testing RDP on all taxonomic levels.

Upper Taxonomic Levels		train0	train1	train2	train3	train4	testing
**genus**	**100 bp**	12150/48650	12150/48650	12150/48650	12150/48650	12200/48600	35750/6350
	**250 bp**	4840/19440	4860/19420	4840/19440	4860/19420	4880/19400	14268/2536
	**500 bp**	2380/9580	2380/9580	2380/9580	2410/9550	2410/9550	6956/1238
**family**	**100 bp**	2300/9350	2300/9350	2350/9300	2350/9300	2350/9300	35440/6300
	**250 bp**	920/3740	920/3740	940/3720	940/3720	940/3720	14136/2520
	**500 bp**	460/1870	460/1870	470/1860	470/1860	470/1860	6890/1242
**order**	**100 bp**	800/3400	850/3350	850/3350	850/3350	850/3350	36760/2280
	**250 bp**	320/1360	340/1340	340/1340	340/1340	340/1340	14668/904
	**500 bp**	150/670	170/650	170/650	160/660	170/650	7172/414
**class**	**100 bp**	550/2400	600/2350	600/2350	600/2350	600/2350	35600/11370
	**250 bp**	220/960	240/940	240/940	240/940	240/940	14208/4532
	**500 bp**	110/470	110/470	120/460	120/460	120/460	6948/2236
**phylum**	**100 bp**	350/1500	350/1500	350/1500	400/1450	400/1450	42070/30
	**250 bp**	140/600	140/600	140/600	160/580	160/580	16792/12
	**500 bp**	70/300	70/300	70/300	80/290	80/290	8188/6

Due to the unbalanced nature of the data, it was difficult to only train on particular genera while also having representatives in the test dataset. Table 2 represents the reads (subsequences) used to train the detector, while Fig. 2 shows the dataset taxonomic composition. A random half of the sequences (4211 16S rRNA sequences) was selected for the detector- training half of the dataset, which was composed of 1216 genera (71% of the genera). After all the restrictions on the training dataset,, the resulting test dataset contained 85% known reads and 15% novel reads on the genus-level.

**Figure 3:**
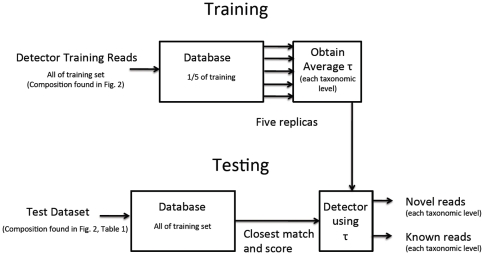
Illustrating how **Figure 2** relates to the overall detector development and testing for each method.

The detector was designed by using 1/10 of the entire data (1/5 of the training dataset) at a time to derive the threshold to be used for the detector. Then, the detector was benchmarked by using the test dataset; the sensitivity, specificity, and their harmonic-mean (the f-measure) were used to measure how well the detector could identify known and novel sequences. An illustration of the detector development and testing is illustrated in Fig. 3.

### II. Detector threshold determination

To develop the detector, we average the thresholds over 5-fold subdivisions of the training dataset (seen in Fig. 3), then the detection threshold was evaluated on the testing set. To develop a detection threshold, we use a method similar to the one outlined in [19] (where Rosen et al. developed a detection threshold for whole genomic data and not just 16S). First, we created a ROC (receiver-operating characteristic) curve using the scores of the RDP, NBC, and Phymm(BL) separately on the training set. Each score was associated with the binary decision of whether the genus exists in the training set or not. The best operating point for each training set was determined as the threshold that obtained the best combined sensitivity and specificity, defined as the maximum point of the f-measure (or the harmonic mean of the sensitivity and specificity). The development of the detector is summarized as follows:

1. Acquire training sequences outlined in part I of the Methods section.
2. Train the scoring method (RDP/NBC/Phymm(BL)) on each 1/5 subset of the training sequences.
3. Construct a read set for the training subset (Table 2) composed of L-length reads simulated from the reserved 4/5ths of the training sequences (Fig. 2), where L is 100 bp, 250 bp, and 500 bp.
4. Score the L-length reads (using RDP, NBC, PhymmBL).
5. Construct an ROC curve using the algorithm's scores and known/unknown labels of the reads.
6. Determine best operating point by maximizing the f-measure.
7. Select the scoring method's threshold corresponding to the best operating point for the training data (to be subsequently used on test data).

### III. Methods used for algorithm comparison

Three scoring methods were used to score reads and we tested how well they worked after selecting a detection threshold. In addition, for RDP, two variants of the scores (the bootstrap and likelihood scores) were tested. Also, in PhymmBL, besides using our detection framework, we also compared against their provided confidence score and selected two scores (80 and 85%) that would be expected to give good sensitivity and specificity. Finally, we also benchmarked against PhylOTU which is a clustering method that does not provide scores.

#### a. RDP bootstrap score

The RDP Classifier (RDP) is based on a naïve Bayes model [11], which assigns a sequence to the closest match using a posterior score. The unique advantage of RDP is that it also provides a bootstrap confidence score, which is able to give a level of confidence to the assignment. This bootstrap score is obtained by taking a random 1/8th of the query (input read) and “reconstructing a new query fragment” then classifying it via the naïve Bayes classifier, iterating this procedure 100 times, and calculating how the proportion of times that the random subset resulted in the same taxon as the original match. This is a way to gauge how susceptible the sequence's classification is to error and incomplete data, etc. If the bootstrap score is low, this means that the sequence may not be from a known taxon and could represent a novel organism.

#### b. NBC and RDP likelihood

When computing the naïve Bayes classification, Bayes theorem derives the posterior probability from conditional and prior probabilities [11], [20], [21]. For this application, the marginal probabilities are assumed to be equiprobable, thereby implying that the likelihood probability is maximized when the posterior probability is maximized [11], [21]. Therefore, the likelihood probability of a read against each genome in the database is computed, and the genome corresponding to the maximum probability is the maximum likelihood solution. This maximum likelihood probability can be interpreted as the probability of the taxon given the read.

#### c. Phymm/PhymmBL's built-in confidence scores

A different phylogenetic classification method, which also learns the underlying sequence composition, is Phymm, which is based on interpolated Markov models (IMMs) [17]. This method is further enhanced in PhymmBL by integrating the IMM probability score with the BLAST score. Since PhymmBL was shown to have better performance than BLAST, we use the hybrid PhymmBL as an “alignment-based” method comparison. The PhymmBL package also produces confidence scores that they recommend to users to differentiate known from novel. For our studies, we trained the detection thresholds using Phymm and PhymmBL scores using the 5-fold training process (see Fig. 3). In addition, we compared the performance of PhymmBL's recommended confidence scores (selecting reasonable thresholds of 80 and 85%).

#### d. PhylOTU

PhylOTU aligns query reads with a database of SSU-rRNA's and then develops a hierarchical clustering with FastTree [18]. With our detection method, we determine if reads are novel or known, while PhylOTU groups the reads, which potentially offers more information since there is a read grouping. In order to benchmark PhylOTU performance at known/novel discrimination, we determined that if PhylOTU clustered the reads with a sequence from the database, the reads are “known”, whereas, if PhylOTU clustered reads in groups without a training representative, they are novel.

### IV. Methods for 16S sequence similarity

We also examined the intra-genus similarity to study the effects on class similarity to novelty detection performance. We used CD-HIT [22] with a similarity threshold of 95% to identify genera that may be too diverse. Using this measure, 76% of the genera contained one cluster, while 92% of the genera contained 3 or fewer clusters. Bacillus was the most diverse genus and contained 50 clusters based on the 95% criterion.

## Results

In this section, we benchmark various methods for their ability to determine which reads are from known taxa (those in the training database) and those reads which are of novel (to the database) origin. We use common metrics to benchmark performance: sensitivity (TP/(TP+FN)), specificity (TN/(TN+FP)), and the f-measure (2*sensitivity*specificity/(sensitivity+specificity)). Essentially, sensitivity measures how well the detector is able to identify “known” reads, while specificity measures how well the detector can identify “novel” reads. Since we would like an optimum of both, we use the harmonic mean of the two, the f-measure to reveal a combined performance.

First, we compare various methods on the genus-level and show that RDP performs the best and compare RDP's performance on higher taxonomic levels. We compare the effects of using only well-represented and higher-similarity genera. Then we test our methods on a sequence data from Amazon soil and demonstrate the computational times of the methods.

### I. Comparison of methods for known/novel detection at the genus-level

First, we show that the RDP classifier has high assignment accuracy for sequences from known genera. Next we explore how well the thresholds from the training set operate on the test set, and finally, we benchmark how well the methods work for known/novel detection.

#### a. Accuracy of RDP classifier for known classification

Before we examine the performance of novel/known detection for RDP, we benchmarked RDP's performance for classifying known taxa. The whole dataset and all reads were used for this benchmarking. For classifying known taxa, the classifier has 90% and above accuracy with near-perfect accuracy for the class-level and above for 250 bp and longer reads (shown in Table 4).

**Table 4:** RDP's accuracy of correct assignment when the sequence that the read originated from was indeed in the training set.

	Genus	Family	Order	Class	Phylum
**100 bp**	90.3+/−0.9%	93.9%+/−0.7%	94.2+/−1.4%	99.1+/−0.5%	99.9+/−0.0%
**250 bp**	98.1+/−0.4%	97.4+/−0.6%	95.2+/−1.3%	99.3+/−0.5%	100+/−0.0%
**500 bp**	99.4+/−0.2%	97.6+/−0.6%	95.2+/−1.4%	99.3+/−0.5%	100+/−0.0%

#### b. ROC analysis (RDP vs. PhymmBL vs. NBC) + detector for novel/known discrimination

**Figure 4:**
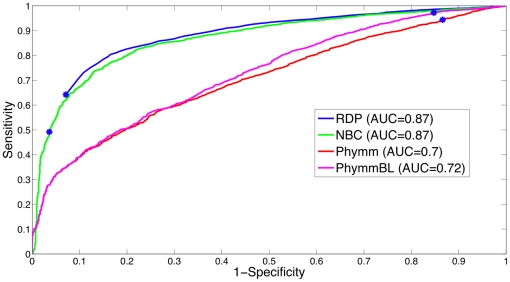
The ROC curve for 4 different novel/known detection methods using the 500 bp read test dataset at the genus-level. The naïve Bayesian methods perform better (higher AUC) than Phymm(BL). The threshold (f-measure) determined chosen from the training data is shown with a blue dot.

**Figure 5:**
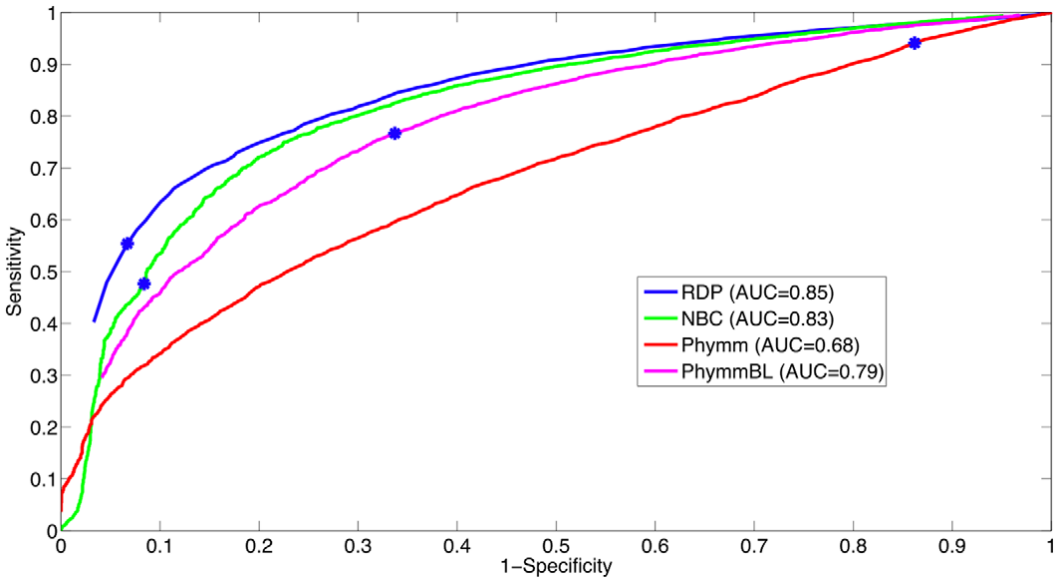
The ROC curve for 4 different methods for 250 bp reads on the genus-level. RDP obtains the best AUC followed by NBC, PhymmBL, and Phymm. The blue star indicates the threshold determined from the training data. In this case, for PhymmBL, the training data determination of the threshold resulted in the most optimal point for the test set unlike the other methods. This results in PhymmBL's good performance in Fig. 8 (bar graph for 250 bp).

**Figure 6:**
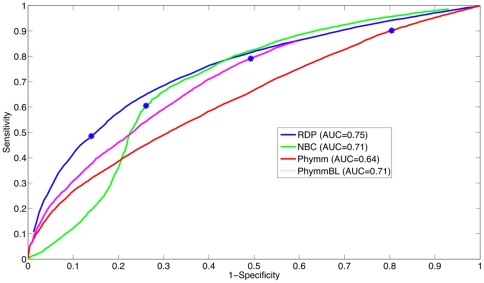
The ROC curve for 4 different methods for 100 bp reads on the genus-level. RDP obtains the best AUC followed by NBC, PhymmBL, and Phymm. The blue star indicates the threshold determined from the training data. In this case, for NBC, the training data determination of the threshold resulted in the most optimal point for the test set unlike the other methods. This results in NBC's good performance in Fig. 16{9} (bar graph for 100 bp).

To develop the detector, we average the threshold over 5-fold subdivisions of the training dataset (seen in Fig. 3), then the detection threshold was evaluated on the testing set. A receiver-operating characteristic curve for the test set on the genus-level is plotted in Fig. 4. The optimal threshold that was chosen through the training procedure, illustrated in Fig. 3, is shown on the ROC curves with a blue dot. In Fig. 4, the ROC area under the curves (AUCs, which can be interpreted as the method's potential) are similar for RDP and NBC, while Phymm and PhymmBL also perform similarly to each other, with the RDP and NBC pair producing the greater/better AUC values. For shorter read lengths (100 and 250 bp) in Figures 5 and 6, RDP maintains its good performance (high AUC) while NBC drops. Also in Fig. 4, we see that at the optimal threshold, both RDP and NBC sacrifice some sensitivity to achieve higher specificity but less so for RDP. In Phymm and PhymmBL, the opposite is true with these methods sacrificing specificity to obtain better sensitivity. For all fourmethods methods, this implies that the training data were not sufficiently diverse to determine an appropriate generalized threshold. Due to limitations in the training data, we can see that the naïve Bayesian methods both obtain low sensitivity/high specificity on the training data while the Phymm-based methods obtain high sensitivity/very low specificity. However, the AUC measure demonstrates that the naïve Bayesian methods have a greater potential on the test dataset, and thus have greater potential as more representatives of rare taxa become available, so the training data becomes more balanced.

#### c. Sensitivity, Specificity, and F-measure of Novel/Known Detection using: RDP Bootstrap, RDP Likelihood, NBC Likelihood, Phymm Score, PhymmBL Score, PhymmBL Confidence Score > = 0.8, PhymmBL Confidence Score > = 0.85

**Figure 7:**
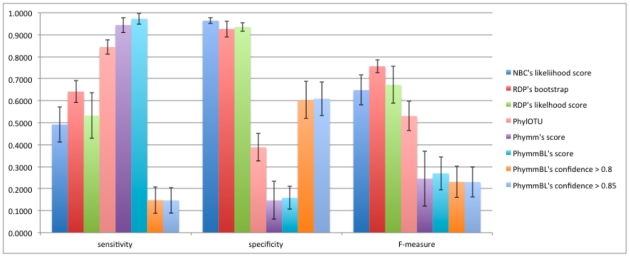
The sensitivity, specificity, and f-measure comparison of novel/known detection of the 500 bp read test dataset on the genus-level. RDP's bootstrap performs the best for being able to discriminate between reads from known and novel origin, with around 76% for the combined f-measure.

**Figure 8:**
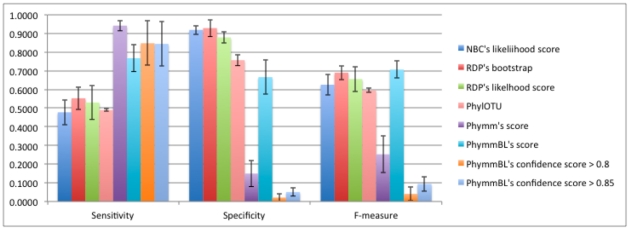
The sensitivity, specificity, and f-measure comparison of 250 bp reads on the genus-level. The naïve Bayesian methods and the hybrid PhymmBL (with empirically chosen threshold) have the best f-measure while Phymm and PhymmBL's built-in confidence measures do not do that well. PhylOTU discarded 789 reads out of 16804 reads. Only those classified are calculated in our performance metric.

**Figure 9:**
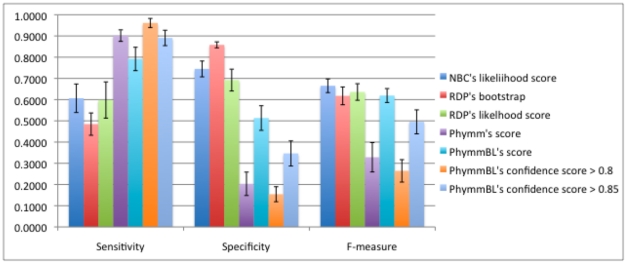
The sensitivity, specificity, and f-measure comparison of 100 bp reads on the genus-level. The Naïve Bayesian methods and the hybrid PhymmBL (with empirically chosen threshold) have the best f-measure while Phymm and PhymmBL's built-in confidence measures do not perform well overall. PhylOTU had memory errors when placing the ∼6400 reads in the test dataset and therefore, there is no performance metric here.

Using the thresholds derived from the process in Fig. 3, we evaluated the process on NBC/RDP's likelihood scores and Phymm/PhymmBL's raw scores. We also evaluated how well the chosen PhymmBL confidence scores and the PhylOTU method performed on the test set; for the latter, we used the training set, but did not go through the 5-fold training shown in Fig. 3. The results of the methods on the test dataset are shown in Fig. 7 for 500 bp reads (with 250 bp and 100 bp reads in Figs. 8 and 9 respectively).

### II. RDP bootstrap at all levels

**Figure 10:**
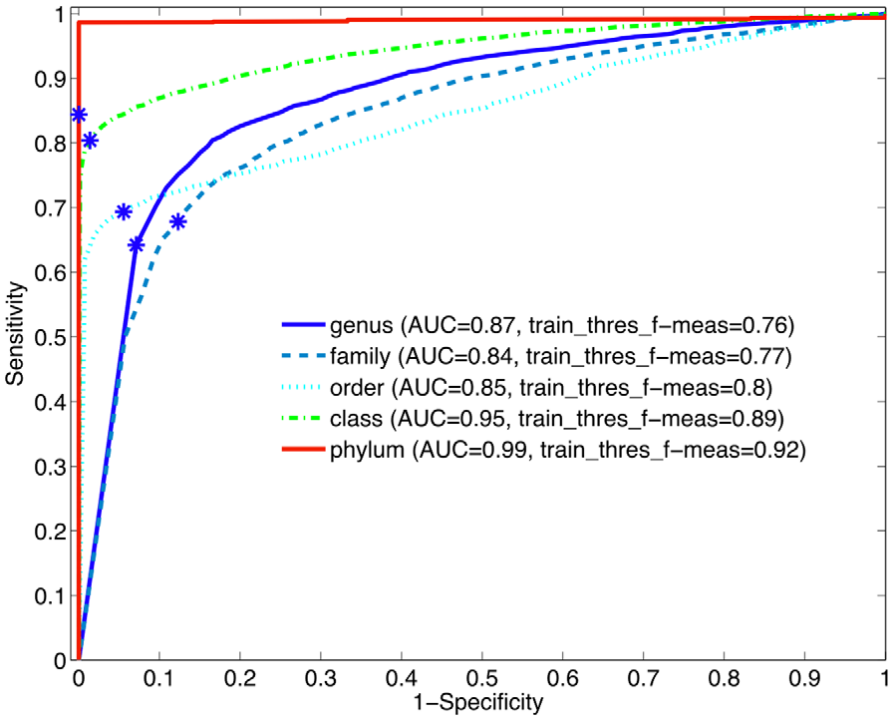
The Receiver-Operating Characteristic Curves for RDP on various taxonomic ranks for 500 bp reads. The Phylum-level has almost perfect performance (maximized TPR while minimized FPR). Surprisingly, family and order have slightly lower AUC than genus, but this is most likely due to taxonomic anomalies at these levels. Using the threshold derived on the training data, the performance on the test data is shown with a blue star.

**Figure 11:**
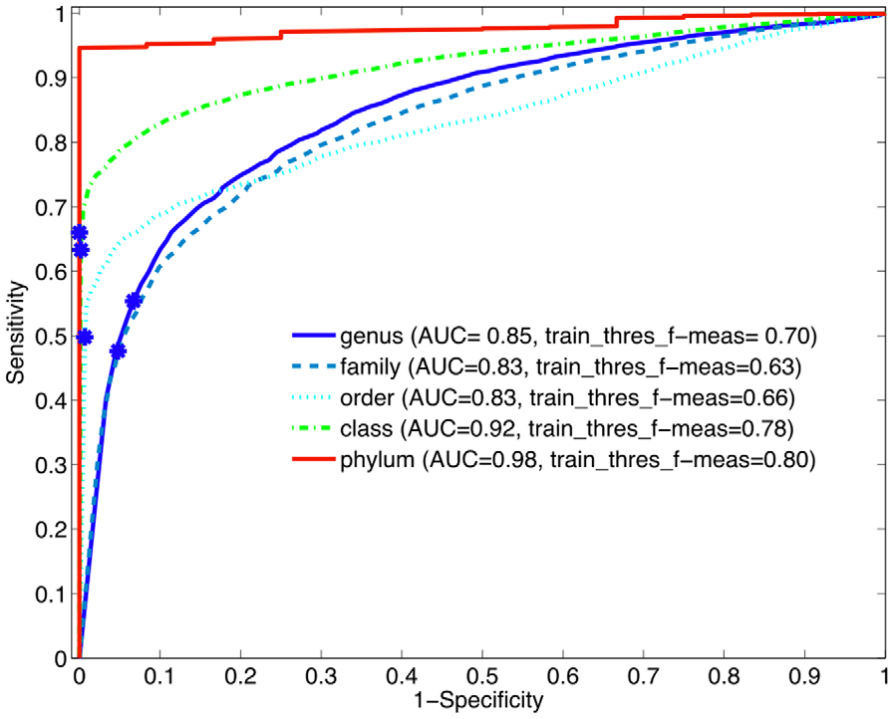
The ROC curve for RDP on all levels for 250 bp reads. Again, the phylum level has high sensitivity at very high specificity.

**Figure 12:**
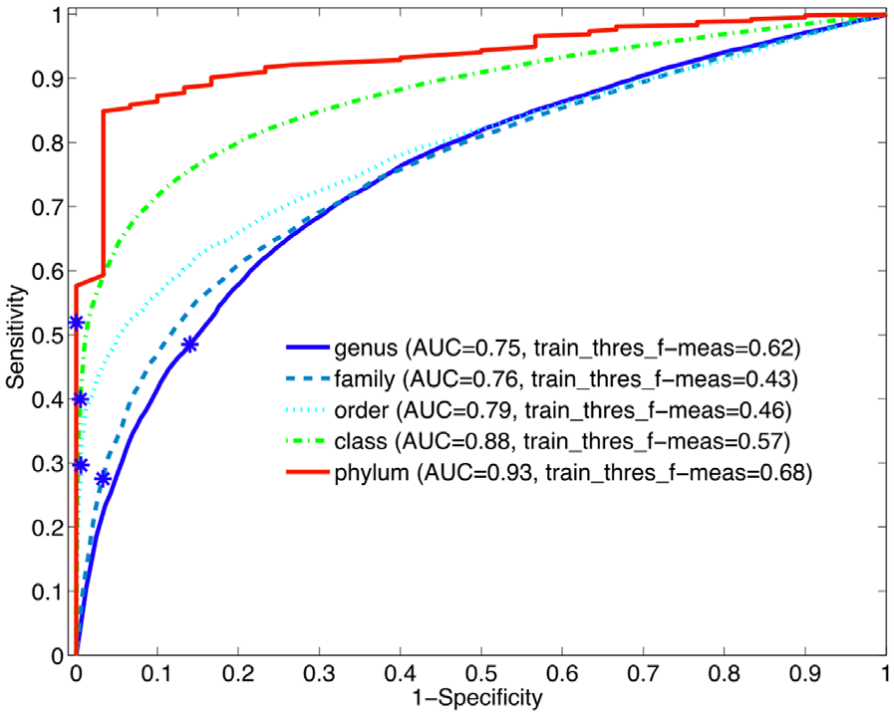
The ROC curve for RDP on all levels for 100 bp reads. Performance decreases for all levels compared to the 500 bp reads but the area-under-the-curves are still over 75%.

Using different training datasets on all levels (see Appendix Tables 3 and 1), we tested the ability of the RDP bootstrap score to predict novel/known reads at three different read-lengths. The bootstrap score yielded a superior operating threshold and F-measure (Table 5) and ROCs (Fig. 10, Fig. 11, and Fig. 12). For 500 bp reads in Fig. 10, the threshold does very well at the at phylum level (AUC of 92%) but not as well at the genus-level (AUC of 76%), with the intermediate taxonomic ranks performing in-between. Either due to some taxa missing labels at particular levels like family and order, or because taxa at these intermediate ranks are on average less phylogenetically coherent than the phylum and genus levels, the performance is not as high as genera for these levels.

**Table 5:** The Sensitivity, Specificity, and F-measure for different read-lengths comparing novel-known detection at genus-level and higher (where each rank is trained separately).

	Read-length	Sensitivity	Specificity	F-measure	Bootstrap threshold
**genus**	100 bp	48.5%+/−0.6%	86.0+/−0.8%	62.0%+/−0.5%	0.7540
	250 bp	55.4%+/−1.2%	93.3%+/−0.7%	69.5%+/−1.1%	0.9640
	500 bp	64.2%+/−1.7%	92.9%+/−1.5%	75.9%+/−1.2%	0.9960
**family**	100 bp	27.6%+/−0.7%	96.7%+/−0.4%	42.9%+/−0.7%	0.8460
	250 bp	47.6%+/−1.1%	95.2%+/−0.7%	63.5%+/−1.2%	0.9800
	500 bp	67.8%+/−1.1%	87.7%+/−1.3%	76.5%+/−1.3%	1.0000
**order**	100 bp	29.7%+/−0.4%	99.4%+/−0.3%	45.7%+/−0.4%	0.9020
	250 bp	49.8%+/−1.3%	99.3%+/−0.4%	66.3%+/−1.3%	0.9980
	500 bp	69.4%+/−0.9%	94.4%+/−1.1%	80.0%+/−0.8%	1.0000
**Class**	100 bp	40.0%+/−1.1%	99.5%+/−0.3%	57.0%+/−1.1%	0.9240
	250 bp	63.3%+/−1.0%	99.8%+/−0.1%	77.5%+/−0.9%	0.9960
	500 bp	80.4%+/−0.6%	98.6%+/−0.2%	88.6%+/−0.4%	0.9960
**phylum**	100 bp	51.9%+/−0.5%	100.0%+/−0%	68.4%+/−0.5%	0.9300
	250 bp	66.0%+/−0.6%	100.0%+/−0%	79.5%+/−0.5%	0.9920
	500 bp	84.4%+/−0.8%	100.0%+/−0%	91.5%+/−0.5%	0.9980

While the sensitivity is low, especially for 100 bp reads, the specificity is high (shown in Fig. 12). This means that at this threshold the detector incorrectly identifies about 50% of the known organisms as novel while correctly identifying all truly novel organisms. In our test set, 85% of the test reads come from known organisms – the method was able to identify almost all of the 15% of novel reads plus filter out over 50% of the known reads, resulting in an approximate reduction of 45% from the potentially novel set! A user can use this as a first step to reduce the search space for novel organisms. For 500 bp reads at order-and-above taxonomic ranks, the f-measure was above 80%, demonstrating that it could identify known from novel reads reasonably well. From the ROC area-under-the-curve metrics for the different levels in Fig. 10, we see that the phylum and class levels have the greatest potential with 99%/95% AUC. For the order-level and below, the curves are not quite as good but reasonable. In Section IV, we directly use the thresholds in our computations on a real dataset to determine how well the detector predicts novel taxa.

### III. Improving classifier performance via well-represented and highly-similar genera

In Fig. 1, we show that the training set is greatly unbalanced. About 50% of genera are represented by only a single sequence, which is possibly the greatest source of poor performance, a conjecture we test in this section for RDP. Also, we hypothesize that using genera where all members are very similar to each other can improve performance – in this case, it did not. In order to illustrate these points, we conducted a “well-represented” and “tightly clustered” training set simulation (see Appendix for details on training/testing sets).

**Figure 13:**
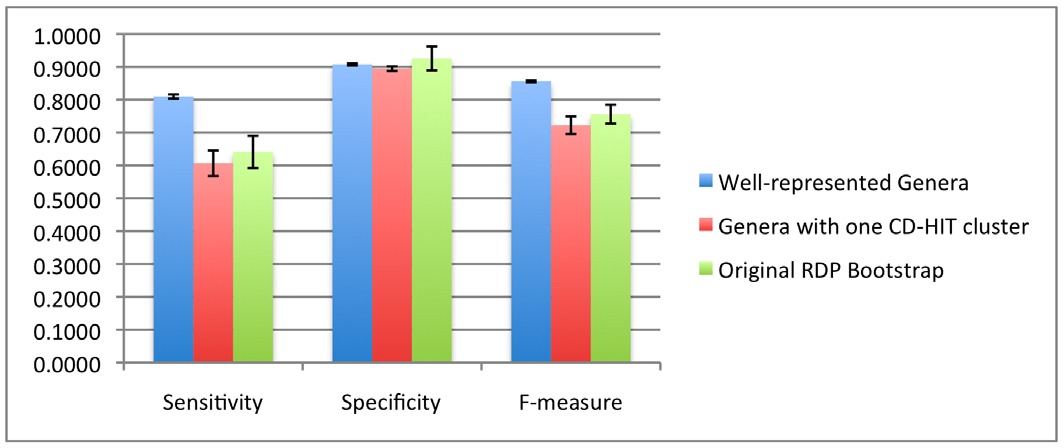
Comparison of 500 bp read performance on databases composed of genera that have at least 10 example sequences (well-represented genera) and genera which have highly similar sequences (Genera with one CD-HIT cluster). While the database with the highly similar genera has about the same performance as the original, the database with the well-represented genera performs about 10% better, in terms of f-measure.

**Figure 14:**
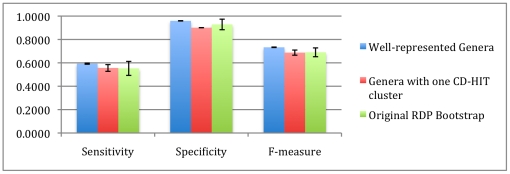
Comparison of 250 bp read performance on databases composed of genera that have at least 10 example sequences (well-represented genera) and genera which have highly similar sequences (Genera with one CD-HIT cluster).

We can see in Fig. 13 that in the case of the well-represented genera, sensitivity rises by 15% and the harmonic mean f-measure rises by 10%. This is due to the fact that new sequences are more confidently assigned and increase the number of true positives. This improvement is across all read lengths (Table 6). This performance improvement is because RDP has more examples to fully learn the genus and therefore makes fewer mistakes marking known organisms (since those genera that are known are well characterized). The well-represented advantage is also maintained at 250 bp, shown in Fig. 14.

**Table 6:** % average increase in performance for different read lengths, using “well-represented” genera that have at least 10 example sequences.

	Sensitivity (% change)	Specificity (% change)	F-measure (% change)	AUC	threshold
**100 bp**	3.7%	6.1%	4.8%	83.6%	0.8740
**250 bp**	4.1%	3.1%	4.3%	90.6%	0.9940
**500 bp**	16.8%	−1.8%	10.0%	91.4%	0.9980

The bootstrap threshold used for the detector is also given.

**Figure 15:**
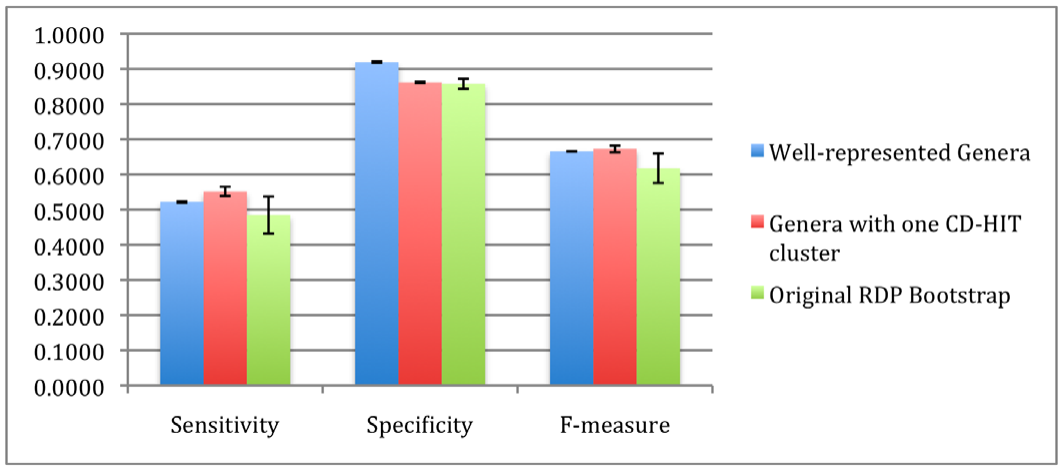
Comparison of 100 bp read performance on databases composed of genera that have at least 10 example sequences (well-represented genera) and genera which have highly similar sequences (Genera with one CD-HIT cluster). The optimal detection threshold determined on the training dataset is shown with a blue star.

In the case of the “tightly clustered” genera experiment (where we only retained genera that met a criterion of clustering with CD-HIT at 95% or higher sequence similarity), performance slightly decreased. While we had thought that tighter groups would be easier for the classifier to “learn”, such a selection mostly retained genera with only one sequence. Therefore, improving the membership of a genus in the training dataset is more important than the intra-genus similarity. For 100 bp reads, both factors (the representation and similarity) help in known/novel detection (seen in Fig. 15).

### IV. Example on a real Metagenomic dataset collected from Amazonian soil

An amazon terra preta soil dataset [23] was obtained from the short read archive (SRA), accession number ERR023723. For this dataset, our goal was to measure how the detector's prediction of novel taxa correlates with the change in number of assignments to a known taxon when the training set size is doubled. The reasoning behind our performance metric is that reads from novel organisms are more likely to find a close match in the “updated” and doubled training dataset and effectively “defect” from the previously known taxa to new taxa added to the new database (or taxa that more closely approximate the “best match”). We find that this occurs for the genus and family levels, where newer taxa are more frequently added. For higher levels such as class and phylum, it is more likely that new examples added to these already existing taxa are the closer match. Therefore, we measure the Pearson correlation of the number of reads assigned to a taxon with half-the-database-plus-detector to how many reads the taxon retains when the full training database is used.. We also find that low abundance of a known taxon can introduce noise into the novelty prediction, since the few that may be marked as novel may be by chance, and we show better correlations for abundant taxa.

**Figure 16:**
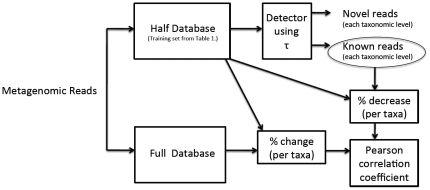
Calculation of the correlation between 1) the detector prediction using the “present” and 2) the full, “future” database. The percent change in the taxon bin is correlated to the previous prediction of novelty of the reads in that bin.

Because the original half-database was used for training, we chose the RDP bootstraps according to the guidelines we recommended in Table 5, adjusted slightly for 230 bp average reads (instead of 250 bp). The RDP bootstrap thresholds chosen are 0.8 for the phylum level, 0.73 for the class level, 0.7 for the order level, 0.74 for the family level, and 0.94 for the genus-level. The process by which we used these thresholds and measured performance on Amazonian dataset is shown in Fig. 16. The Amazonian read dataset was sent through the RDP classifier trained on the half-database. Then the RDP bootstrap scores were processed by the detector. We repeat the process using the full training database and measure the relative percent drop in novel reads (from the half-database predictions) for each taxon.

**Figure 17:**
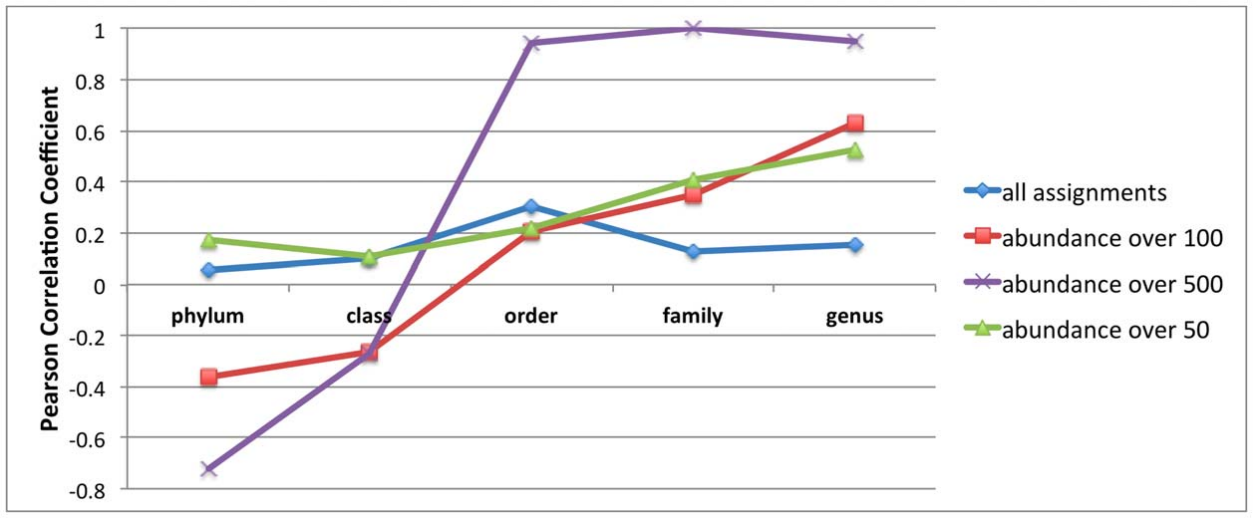
Pearson correlation coefficient of the decrease predicted by the detector (from the half-database) to the change in abundance in that taxa with the full, updated database for an amazon soil pyrosequence dataset.

The best correlations were for highly abundant taxa (predicted by the original half-database; Fig. 17). The detector predicted some taxa may attract more novel reads than others (by the # of reads that passed the detector), and this was directly correlated with the fraction of reads that stayed or defected from that taxon when more examples were added with the full-database. We based our correlations upon the relative decreases, since reads in a truly novel taxon may not “defect” as much as we would hope, since there is no better match in the database (Table 7). In fact, some phyla now contain a better match for reads that were incorrectly classified to known phyla before, and this introduces an inverse correlation on the class and phylum levels (seen in Fig. 17) due to the fact that these “known” taxa are missing many representative examples (since there are few novel classes/phyla). In Table 7, we see that there is a correlation between the amount that the detector passes as “known” to the amount classified in the full-database.

**Table 7:** The abundance numbers after each step in Fig. 8{16}.

Order	Half	Half+ Detector	Full	Family	Half	Half+ Detector	Full	Genus	Half	Half+ Detector	Full
Rhizobiales	1092	1040	1112	Conexibacteraceae	757	45	646	Spartobacteria_genera_incertae_sedis	1086	987	1082
Solirubrobacterales	821	188	705	Hyphomicrobiaceae	554	249	523	Conexibacter	757	7	646
Actinomycetales	648	466	596					Gp6	676	468	674
								Gp1	507	326	506

The novelty predicted by the detector is correlated to a decrease in abundant (over-500 occurrences) taxa, when using the RDP trained on the full (future) database.

To better illustrate, in Fig. 17, we show the effect of observing the correlation for all taxa classified (blue line), not just the highly abundant taxa. For rarer taxa, it is difficult to measure the relative change, for example, if an RDP bin contains 3 reads, a removal of one will result in a 33% decrease, and if this correlated to 2 defections out of this taxon and when testing against the full-database, this would amount to a 66% decrease. It is harder to measure a linear correlation with such noisy measurements, so there is very little correlation for all taxa. We also show that as we examine the more abundant taxa (those with abundances over-50, over-100, and over-500 assignments), the detector's ability to predict at the genus- and family-levels increases.

### V. CPU time

On a computer with 2 Intel®Core™CPU @ 1.86 GHz Speed and 2 GB of memory, the methods in the paper were benchmarked. In Table 8, we can see that RDP was 20–30 times faster than most methods in training. Most importantly, RDP is 60–140 times faster in testing over other methods. While RDP's time increases as a function of the read-length, NBC/PhymmBL/PhylOTU's time decreased because the number of test reads in each dataset decreased, as shown in Table 2.

**Table 8:** Runtimes of the various methods (in minutes).

	Training	100 bp Test Set	250 bp Test Set	500 bp Test Set
RDP	0.22	1.57	1.67	1.69
NBC	0.35	132.21	112.10	100.91
PhymmBL	4.73	165.33	147.30	138.51
PhylOTU	6.18	N/A	239.51	139.70

While RDP provides the best known/novel performance, it is also the fastest.

## Discussion

Through rigorous benchmarking, we find that developing a threshold based on the RDP bootstrap score results in the best novelty detection performance of the detectors tested, with an f-measure (a harmonic mean of sensitivity and specificity) of 75% and higher for 500 bp reads. It is very conservative in its novel detection, in that it detects novel reads almost perfectly (high sensitivity), but also tends to mark reads from known organisms as novel (low specificity). If the user wishes to reduce the search space of reads from novel taxa in dataset, this detector would be an easy and fast first filtering step. In low complexity samples, such as the oral cavity [24], where most taxa are well-known, this could constitute useful method to identify those reads that originate from novel organisms. For complex samples, such as soil, this method can act as a filter to identify a smaller set of reads that may come from novel taxa, thus reducing the time it may take to run binning algorithms (such as PhylOTU) on these sequences. While RDP does perform well, we have found that the bootstrap score is read-length dependent. If 1/8 of the sequence is used for bootstrapping, more basepairs will determine the bootstrap percentage for longer reads. Since the bootstrap score is read-length dependent, we recommend using a standard number of basepairs (such as 100 bp out of any sequence length) chosen randomly to compute the bootstrap. This would result in the input minimum read length to be at least 200–300 bp (in order to choose this 100 bp subset).

We show that a 10% improvement (for 500 bp reads) can be achieved just by increasing the number of training sequences to at least 10 per taxon. The more training data available to a taxa, the better the classification performance that can be achieved. This places great importance on projects such as the Genomic Encyclopedia of Bacteria and Archaea (GEBA) [25], which aim to sequence novel and ill-represented taxa.

A comparison of all the methods on the test dataset is shown in Fig. 7. We chose to benchmark the methods using the sensitivity, specificity, and harmonic mean of the two, since sensitivity determines how well the detector can sense “known” reads, specificity determines how well the detector can sense “unknown” reads, and the F-measure is an equally weighted balance between the two. We can see that the naïve Bayes classification methods perform similarly, with RDP's bootstrap score outperforming other methods for the f-measure. The RDP optimal bootstrap threshold is 99.6%. The Phymm/PhymmBL methods showed high sensitivity but very low specificity (using our detector method) resulting in a low f-measure. Reasonable thresholds for PhymmBL's built-in confidence metric (80–85%) performed similarly to the PhymmBL raw score in terms of f-measure. (A 90% confidence threshold performed even worse.) For PhylOTU, 1502 out of 8194 reads (in the 500 bp test set) were discarded by the algorithm (due to its quality filters, etc.), and only the reads that were not discarded were measured in the performance metric in Fig. 7. For PhylOTU's results, if a read clustered with any sequence in the training dataset, it was counted as “known” whereas if it clustered by itself or with other reads not in the training dataset, it was counted as novel. In conclusion, the two Bayesian methods gave more balanced results, with RDP using the bootstrap performing somewhat better overall in discriminating reads from novel and known taxa.

We would like the reader to note that our detector threshold was trained using only half the standard data for training and carving this dataset into five subdivisions, each using 1/5 of ½ of the RDP classifier database. But in each training iteration, all training dataset reads are used – 1/5 are from examples of known genera and the other 4/5 are from novel genera. This simulates a scenario where the detector threshold is trained on samples where 20% of the reads originate from known-to-the-database genera. This training of detector using1/5^th^-known and 4/5^th^-novel data may account for the conservativeness (# of FNs >> # of FPs) of the detection threshold. In reality, the known/novel composition of the sample may vary (20% could be low for the oral cavity but high for soil). So, we provide this as a caution for those processing samples that may be low-complexity and/or have many organisms that have been previously sequenced – some reads from known organisms will be falsely labeled as novel. We recommend the bootstrap thresholds shown in Table 5 be used as a rough guideline for interpreting RDP classifier results.

We demonstrate on a soil dataset that if the detector only passes a fraction of reads for a particular taxon, when trained on the half-dataset, the reads assigned to that taxon decrease when the full (doubled) database is used for training. This shows that the detector was able to successfully predict that those reads most likely originate from novel organisms. Of course, if there is no better match in the database or no novel taxa at high phylogenetic levels (such as class or phylum), the reads will still be match to the same incorrect taxa but with low bootstrap scores. For highly abundant taxa, where there is more diversity in reads, we can show that those reads predicted as novel do “defect” to better matching taxa when the full dataset is used for training, thereby showing the efficacy of the novelty detection.

There are several main conclusions for the increase in correlation from phylum to family and genus in Fig. 17. For highly abundant taxa, the more reads that the detector predicts as novel (with the half-database), the higher the decrease in that taxon (on the order-, family-, and genus-levels) after classification using the full-database. It is more likely that the closest match is a newly added member of a known phylum (or class) and therefore, this trend inverts, since the database gets few added phyla or classes in the updated database; the trend is due to the fact that reads from novel taxa are still being incorrectly matched to known taxa, not to the fault of the detector but to the fact that the true phylum/class has not been added to the database yet. Also, the less abundant taxa are more difficult to assess since the “relative proportion calculations” are noisy. Nonetheless, for genera bins that contain over 50 reads, the Pearson correlation coefficient is about 0.5 which still shows a significant linear correlation.

While detecting whether a read is from “known” (in the training data) or “novel” origin is a challenging task with such little “known” data, we show in our study that classification methods can discriminate between reads originating from known and unknown organisms. By carefully selecting a threshold using the current database, the RDP classifier and its corresponding bootstrap score can offer a known/novel assignment better than most methods. It is a quick method that does not rely upon alignment or BLAST. We show that the method is highly conservative in its identification of reads from known taxa. Therefore, we recommend that the RDP bootstrap can be used as a first step to isolate reads from novel genera and can reduce the search space significantly if the sample contains many reads from known taxa. The next step after this would be to perform alignment to determine a sequence's phylogenetic placement, such as the SOPPI protocol [26] or to group the “novel” reads to determine which belong to the same taxonomic groups. Programs such as PhylOTU can be used for this purpose, and we recommend using the RDP bootstrap score for known/novel detection to enhance the PhylOTU's tendency to discard reads and to add confidence to clustering of reads.
